# Operating Microscope in the Analysis of Localized Juvenile Spongiotic Gingival Hyperplasia

**DOI:** 10.1111/odi.70031

**Published:** 2025-07-13

**Authors:** Yuri de Lima Medeiros, João Batista César Neto, Fabio Abreu Alves

**Affiliations:** ^1^ Department of Stomatology, School of Dentistry São Paulo University São Paulo Brazil; ^2^ Department of Stomatology A.C.Camargo Cancer Center São Paulo Brazil

**Keywords:** gingival lesions, microscopy, spongiotic gingival hyperplasia

## Abstract

The aim of this study was to describe the clinical microscopic features of localized juvenile spongiotic gingival hyperplasia (LJSGH) using an operating microscope (OM), a non‐invasive technique rarely applied to oral mucosal lesions. An 18‐year‐old female presented with asymptomatic gingival redness and bleeding affecting the buccal gingiva of previously orthodontically treated maxillary canines. Conventional clinical examination revealed gingival recession, erythema, and edema. OM evaluation enabled detailed visualization of gingival surface irregularities, superficial vascular spots, exudation, and friable tissue that bled upon touch and probing. An incisional biopsy was performed. Histopathological analysis showed acanthosis, spongiosis, exocytosis, congested capillaries at the epithelium‐connective tissue interface, and a dense inflammatory infiltrate in the lamina propria, confirming the diagnosis of LJSGH. In conclusion, OM allowed the identification of vascularity, exudation, surface irregularity, and tissue friability, features not apparent in conventional clinical examination. These findings suggest that OM is a valuable adjunct for evaluating and documenting gingival lesions.

1

Localized juvenile spongiotic gingival hyperplasia (LJSGH) is a rare entity, occurring most frequently in children and young individuals. It presents as a painless, bright red lesion with a velvety or granular texture. Isolated or multifocal cases frequently affect the gingiva of the maxillary anterior teeth. The pathogenesis is not completely understood, but histopathologic similarities with junctional epithelium and expression of cytokeratin 19 may suggest an odontogenic origin. Lack of keratinization, presence of spongiosis, and exocytosis are other histopathologic findings (Theofilou et al. 2021).

Direct oral microscopy is an innovative non‐invasive technique which may be added to clinical examination of the oral cavity. Some principles are derived from colposcopy and dermatoscopy. Although widely used in endodontics and operative dentistry (Liu et al. 2023), this technique has rarely been applied to oral mucosal lesions. The operating microscope (OM) allows examination of the oral mucosa at multiple magnifications to evaluate features such as lesion limits, subepithelial vessels, surface patterns, color, and transparency (Drogoszewska et al. 2013; Gynther et al. 2000). Despite its benefits, to the best of our knowledge, no other study has performed a clinical microscopic analysis of gingival lesions. The aim of this study was to highlight the clinical aspects of LJSGH observed in the OM.

An 18‐year‐old female presented with complaints of asymptomatic gingival redness and bleeding. Her medical history includes atopic dermatitis treated with topical corticosteroids. On clinical examination, gingival recession, edema, and erythema were observed on the buccal gingiva of both maxillary canines, which had been previously subjected to orthodontic traction (Figure 1).

**FIGURE 1:**
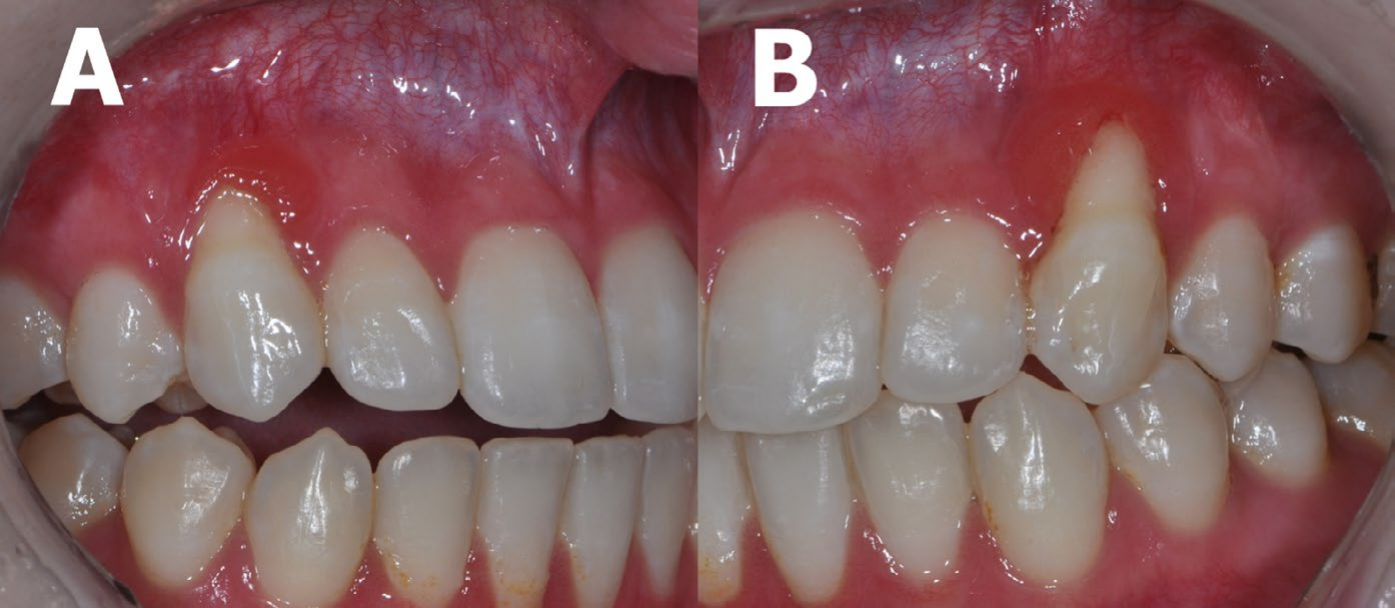
Clinical appearance, demonstrating gingival recession and erythema located in the buccal gingiva of the (A) right and (B) left upper canine.

Gingival details were evaluated using an OM (Decius Standard Microscope, DF Vasconcellos SA, São Paulo, SP, Brazil), which allowed visualization of the irregular gingival surface, superficial vascular points, and exudation. Moreover, the lesions were soft, friable, and bleeding on touch and probing with a periodontal probe (Video S1).

The diagnostic hypotheses were LJSGH and gingival hyperplasia. Incisional biopsy was performed. Histopathological analysis revealed acanthosis, spongiosis, exocytosis, congested capillaries at the epithelium‐connective tissue interface, and a dense inflammatory infiltrate in the lamina propria (Figure 2). According to clinical and histopathological features, LJSGH diagnosis was established. The patient was instructed on oral hygiene and monitored every 4 months for dental prophylaxis. Clobetasol propionate 0.05% has been prescribed only in exacerbation periods. The patient has currently been monitored for 2 years and is in stable condition.

**FIGURE 2:**
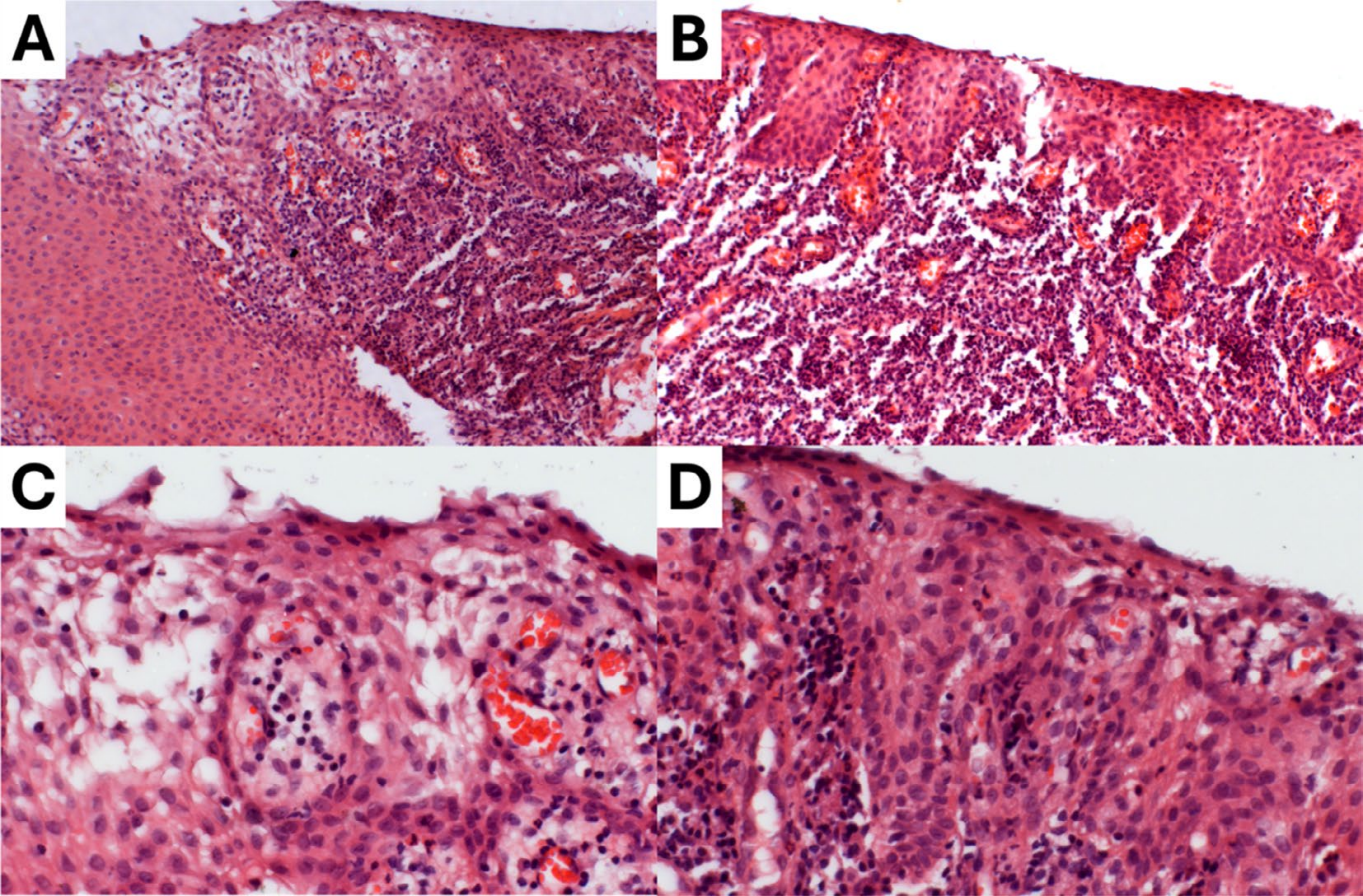
Histopathological features of localized juvenile spongiotic gingival hyperplasia. (A, B) Spongiotic non‐keratinized stratified squamous epithelium, acanthosis and a dense and diffuse inflammatory infiltrate (H&E, ×10). (C, D) Spongiosis, exocytosis, and congested capillaries (H&E, ×40).

Studies using direct microscopy to analyze oral lesions are scarce and have mostly been used to characterize clinical features of oral potentially malignant disorders and surgical margins of malignant tumors (Gynther et al. 2000; Reddy et al. 2022; Shetty et al. 2011). However, as seen in our report, the OM can also be useful in improving the clinical description and analysis of superficial gingival lesions. The OM made it possible to observe the presence of several superficial blood points in our patient. Shetty et al. described different vascular patterns that can be visualized by direct oral microscopy in oral lesions, such as network, hairpin, punctate, mosaic, and atypical capillaries (Shetty et al. 2011). Clinically, through conventional clinical examination, it was only possible to observe the bright red color, due to highly vascularized connective tissue associated with an absence of keratinization (Theofilou et al. 2021).

In addition to vascular changes, the presence of superficial irregularities, exudation, and softness of the tissue are not commonly described as characteristics of this disease and, in the present case, it could be observed by microscopy. Magnification also allowed for a very clear observation of how the exudate emerges from the underlying connective tissue to the epithelium, and this occurred immediately after the surface was dried with air. Furthermore, although the surface is generally described as granular (Innocentini et al. 2020), the magnification observed in this case showed irregularities of the gingival surface, highlighting variations in texture and contour that might be overlooked during a conventional clinical examination. Such details contributed to distinguishing LJSGH from other gingival inflammatory alterations, leading to a more reliable diagnosis.

The OM provides a more accurate assessment of the gingival boundaries involved. Most LJSGH cases have been described affecting the attached gingiva, with a band of healthy tissue separating it from the marginal gingiva (Decani et al. 2021; Theofilou et al. 2021). However, other studies have reported marginal gingiva involvement (Innocentini et al. 2020; Kala and Prasad 2023) as observed in the present case. High magnification may contribute to a more accurate evaluation of marginal gingiva involvement in LJSGH, aiding in the exclusion of other gingival conditions, such as plaque‐related diseases and non‐neoplastic reactive overgrowths.

In summary, vascularity, softness, tissue irregularity, and exudate were gingival details found in the analysis of LJSGH with OM that could not be observed by conventional clinical examination. The OM may be an option for better evaluation and description of superficial gingival lesions.

## Author Contributions

**Yuri de Lima Medeiros:** conceptualization, data curation, investigation, methodology, validation, visualization, writing – original draft. **João Batista César Neto:** conceptualization, formal analysis, investigation, methodology, project administration, validation, visualization, writing – review and editing. **Fabio Abreu Alves:** conceptualization, data curation, investigation, methodology, project administration, supervision, validation, writing – review and editing.

## Ethics Statement

All procedures performed in studies involving human participants were in accordance with the ethical standards of the institutional and national research committee and with the 1964 Helsinki declaration and its later amendments or comparable ethical standards.

## Consent

Informed consent was obtained from the patient.

## Conflicts of Interest

The authors declare no conflicts of interest.

## Supporting information


**Video S1:** Microscopic aspects of localized juvenile spongiotic gingival hyperplasia.

## Data Availability

The data that support the findings of this study are available from the corresponding author upon reasonable request.
